# Cardiac insulin-like growth factor-1 and cyclins gene expression in canine models of ischemic or overpacing cardiomyopathy

**DOI:** 10.1186/1471-2261-9-49

**Published:** 2009-10-09

**Authors:** Maryam Mahmoudabady, Myrielle Mathieu, Karim Touihri, Ielham Hadad, Agnes Mendes Da Costa, Robert Naeije, Kathleen Mc Entee

**Affiliations:** 1 Laboratory of Physiology, Faculty of Medicine, Université Libre de Bruxelles, Brussels, Belgium; 2 Department of Physiology, Faculty of Medicine, Mashhad University of Medical Sciences (MUMS), Mashhad, Iran

## Abstract

**Background:**

Insulin-like growth factor-1 (IGF-1), transforming growth factor β (TGFβ) and cyclins are thought to play a role in myocardial hypertrophic response to insults. We investigated these signaling pathways in canine models of ischemic or overpacing-induced cardiomyopathy.

**Methods:**

Echocardiographic recordings and myocardial sampling for measurements of gene expressions of IGF-1, its receptor (IGF-1R), TGFβ and of cyclins A, B, D1, D2, D3 and E, were obtained in 8 dogs with a healed myocardial infarction, 8 dogs after 7 weeks of overpacing and in 7 healthy control dogs.

**Results:**

Ischemic cardiomyopathy was characterized by moderate left ventricular systolic dysfunction and eccentric hypertrophy, with increased expressions of IGF-1, IGF-1R and cyclins B, D1, D3 and E. Tachycardiomyopathy was characterized by severe left ventricular systolic dysfunction and dilation with no identifiable hypertrophic response. In the latter model, only IGF-1 was overexpressed while IGF-1R, cyclins B, D1, D3 and E stayed unchanged as compared to controls. The expressions of TGFβ, cyclins A and D2 were comparable in the 3 groups. The expression of IGF-1R was correlated with the thickness of the interventricular septum, in systole and diastole, and to cyclins B, D1, D3 and E expression.

**Conclusion:**

These results agree with the notion that IGF-1/IGF-1R and cyclins are involved in the hypertrophic response observed in cardiomyopathies.

## Background

Insulin-like growth factor-1 (IGF-1) is thought to be involved in cardiac tissue maintenance and function. Interaction of IGF-1 with its receptor (IGF-1R) stimulates DNA and protein syntheses [1], contractility [2] and inhibits apoptosis [3] in cardiac myocytes through an autocrine pathway [4]. This signaling pathway also participates in post-ischemic neovascularisation, promoting multiplication of endothelial cells and formation of endothelial tubes[5].

Transforming growth factor β (TGFβ) participates in the cardiac remodeling process including cardiac fibrosis and modulation of cardiac myocytes hypertrophy. In vitro, TGFβ induces the production of extracellular matrix components, stimulates fibroblast proliferation and its conversion to myofibroblast [6]. In vivo, TGFβ is correlated with the degree of fibrosis [7]. The involvement of the TGFβ-activated kinase 1 pathway induces cardiac hypertrophy while the Smad-dependent pathway may inhibit cardiac myocytes hypertrophy [8].

Whether cardiac myocytes might retain the capability to proliferate has remained unsettled until the very recent report of a 1-0.5% yearly rate of replacement of cardiac muscle cells in human adults [9]. In the adult heart, the majority of myocytes are blocked in the G0 or G1 phases of the cell cycle, consistent with terminal differentiation of these cells. However, in vitro studies have shown that the interaction of IGF-1 with its IGF-1R receptor may stimulate the re-entry of adult ventricular myocytes into the cell cycle [10]. These cells therefore proceed through G1 to synthesize the necessary mRNA and proteins for hypertrophic growth and some will enter the S phase and undergo DNA synthesis and binucleation [11] and probably also mitosis in a proportion of them. Cell cycle progression is controlled by regulatory molecules called cyclins [12].

Different types of cardiomyopathies are associated with variable hypertrophic and perhaps hyperplasic responses, and these can be reproduced experimentally. We previously reported canine models of overpacing-induced dilated cardiomyopathy with no detectable hypertrophy [13] and of ischemic cardiomyopathy on a healed small size myocardial infarction with a marked hypertrophic reaction [14]. We therefore investigated the gene expressions of IGF-1 and cyclins in these two models of cardiac disease.

## Methods

All the investigation protocols had been approved by the Institutional Animal Care and Use Committee of the Université Libre de Bruxelles and were conducted in accordance with the Guide for the Care and Use of Laboratory Animals published by the National Institutes of Health (NIH Publication No. 85-23. Revised 1996).

Echocardiographic recordings and myocardial tissue specimens were obtained from 8 beagle dogs 28 weeks after a myocardial infarction, 8 beagle dogs after 7 weeks of overpacing and 7 healthy control dogs.

### Cardiomyopathy secondary to healed myocardial infarction

Briefly, under general anesthesia, a left thoracotomy was performed to ligate directly the left circumflex artery and/or its marginales, as previously described [14]. An echocardiographic examination was performed at baseline and 28 weeks after recovery. The animals were then killed by an overdose of anesthetics, and myocardial tissue sampled from the infarcted and border zones situated in the left ventricular free wall and from a remote zone situated in the interventricular septum. The tissue samples were snap frozen in liquid nitrogen and stored at -80°C until use.

### Overpacing-induced dilated cardiomyopathy

Briefly, under general anesthesia, a transvenous pacemaker lead was inserted in the right ventricular apex and connected to a pacemaker inserted subcutaneously in the cervical region, as previously described [13]. After a 2-week recovery, the dogs underwent a modified pacing protocol with stepwise increases in stimulation frequency. The pacing was initiated at a rate of 180 beats per minute for one week, followed by a rate of 200 beats per minute during a second week, 220 beats per minute during a third week and finally 240 beats per minute from week 4 to week 7. Echocardiography was performed at baseline (before activation of the pacemaker) and at week 7. The animals were then killed by an overdose of anesthetics, and myocardial tissue sampled from the left ventricular free wall and from the interventricular septum. The samples were snap frozen in liquid nitrogen and stored at -80°C until use.

### Control dogs

Myocardial tissue of the left ventricular free wall and of the interventricular septum were harvested from 7 normal beagle dogs killed with an overdose of anesthetics.

### Echocardiography

Echocardiography (Vivid 5, GE, Brussels, Belgium or Pandion, Pie Medical Benelux, Zaventem, Belgium) was carried out under continuous ECG monitoring with a 5 MHZ electronic probe, the dog lying in lateral recumbency being scanned through the dependent chest wall [15]. A bidimensional guided M-mode right short axis view of the left ventricle at the level of the chordae tendinae was recorded. From these recordings, we measured end-diastolic and end-systolic left ventricular diameters (LVDd and LVDs), interventricular septum thickness (IVSd and IVSs) and left ventricular free wall thickness (LVFWd and LVFWs). Left ventricular end-diastolic and end-systolic volumes (LVEDV and LVESV), fractional shorthening (FS), ejection fraction (LVEF), interventricular septum and left ventricular free wall fractional thickening (IVSFT and LVFWFT), relative wall thickness (RWT) and left ventricular mass (LVmass) were calculated using formulas shown on listed in table 1. The mean value of 3 measurements of the technically best cardiac, cycles irrespective of the respiratory phase, was taken. All images were analyzed according to the recommendations of the American Society of Echocardiography [16] and the Echocardiography Committee of the Specialty of Cardiology, American College of Veterinary Internal Medicine [17].

**Table 1:** Formulas which were used for calculating echocardiographic indices

***Echocardiographic indices***	***Formulas***
LVEDV	(LVDd^3 ^× 7)/(2.4 + LVDd
LVESV	(LVDs^3 ^× 7)/(2.4 + LVDs)
LVEF	((EDV-ESV)/EDV) × 100
FS	{(LVDd -- LVDs)/LVDd} × 100
IVSFT	{(IVSs -- IVSd)/IVSd} × 100
LVFWFT	{(LVFWs -- LVFWd)/LVFWd} × 100
RWT	2 × (IVSd/LVDd)
LV mass	1.04 × {(LVDd + IVSd + LVFWd)^3 ^- LVDd^3^}

LVEDV, left ventricular end diastolic volume; LVESV, left ventricular end systolic volume; LVEF, left ventricular ejection fraction; FS, fractional shortening; IVSFT, interventricular septum fractional thickening; LVFWFT, left ventricular free wall fractional thickening; RWT, relative wall thickness; LVmass, left ventricular mass

### Quantitative real time polymerase chain reaction (QRT-PCR)

Total RNA was extracted using the Trizol method (Invitrogen, Merelbeke, Belgium)., After first strand cDNA synthesis, Sybr Green RTQ-PCR was performed (Icycler, Biorad Laboratory, Nazareth, Belgium). For each gene, the PCR conditions were optimized to obtain only the specific product with an efficiency calculated from dilution curves (7×) between 95 and 105%. Each sample was measured in triplicate and each plate contained negative and positive controls. Abbelson (ABL) was used as a housekeeping gene. Statistical analysis was carried out using the differences between the cycles threshold (Ct) (ΔCt = Ct gene of interest - Ct housekeeping gene). Relative gene expression values were obtained using the ΔΔCt method (ΔCt sample - ΔCt calibrator) using control (CTRL) as calibrator. The conversion of ΔΔCt to relative gene expression is Fold induction: 2^-ΔΔCt ^[18].

### Statistical analysis

All results are expressed as mean ± SEM and statistical significance is determined at p < 0.05. Distribution normality was tested with a Kolmogorov-Smirnov test. Echocardiographic data were compared by paired t-tests. For QRT-PCR data, normally distributed data were tested by a one-way ANOVA, followed by Scheffé post-hoc tests when overall significance was detected. When the Kolmogorov-Smirnov test failed, differences between groups were tested by a Kruskal-Wallis test. Coefficients of weighted linear regression (*R*) and associated probabilities (*P*) were determined to examine the relationship between relative cardiac IGF-1R mRNA expression (*x*-axis) and the interventricular septum thickness or relative cardiac cyclins expression (*y*-axis) [19].

## Results

### Echocardiography

Table 2 summarizes the echocardiographic measurements in the dogs with ischemic cardiomyopathy and tachycardiomyopathy respectively, at baseline and before euthanasia.

**Table 2:** Echocardiographic data in postischemic and overpacing models

	***Infarct***		***Paced***	
***Echocardiographic indices***	***Baseline***	***End***	***Baseline***	***End***

LVEF,%	68 ± 3	55.5 ± 3 §	66 ± 2	35 ± 2 *
FS, %	37 ± 2	33 ± 1	36 ± 1	17 ± 1 *
LV mass, g	92 ± 4	118 ± 8 ‡	126 ± 7	127 ± 6
IVSd, mm	7.1 ± 0.2	8 ± 0.3	7 ± 0.2	6 ± 0.3 §
IVSs, mm	11.2 ± 0.4	12 ± 0.8	11 ± 0.6	6 ± 0.3 *
LVDd, mm	36.5 ± 0.5	42 ± 2 §	39 ± 1.5	50 ± 2 *
LVDs, mm	23 ± 1	28 ± 1 §	25 ± 1	41 ± 1 *
LVFWd, mm	8 ± 0.3	7.2 ± 0.5	7.8 ± 0.4	6.5 ± 0.3 ‡
LVFWs, mm	12 ± 0.3	10 ± 0.7 ‡	12 ± 0.5	9.6 ± 0.4 §
RWT	0.4 ± 0.02	0.4 ± 0.02	0.4 ± 0.02	0.2 ± 0.02 *
LVEDV, ml	56 ± 2	81 ± 8 §	66 ± 6	117 ± 9 *
LVESV, ml	18 ± 2	32 ± 4 §	22 ± 2	76 ± 6 *
IVSFT, %	58 ± 7	48 ± 6	49 ± 9	14 ± 4 §
LVFWFT, %	53 ± 4	40 ± 5 ‡	56 ± 3	40 ± 5 ‡

LVEF, left ventricular ejection fraction; FS, fractional shortening; LVmass, left ventriular mass; IVSd, interventricular septum thickness in diastole; IVSs, interventricular septum thickness in systole; LVDd, left ventricular diameter in diastole; LVDs, left ventricular diameter in systole; LVPWd, left ventricular posterior wall diameter in diastole; LVPWs, left ventricular posterior wall diameter in systole; RWT, relative wall thickness; LVEDV, left ventricular end diastolic volume; LVESV, left ventricular end systolic volume; IVSFT, interventricular septum fractional thickening; LVFWFT, left ventricular free wall fractional thickening. ‡ p < 0.05, § p < 0.01, * p < 0.001 in each end group versus baseline.

The dogs with ischemic cardiomyopathy had increased LVEDV, IVSd and LVmass and unchanged RWT, indicating eccentric hypertrophy. The LVEF was decreased but remained above 50%, The FS was mildly reduced but remained above 25% while IVSFT and LVFWFT were also reduced and LVESV was increased, indicating moderate global systolic dysfunction. The dogs with tachycardiomyopathy had increased LVEDV with decreased IVSd, LVFWd and RWT and no change in LVmass indicating LV dilation without hypertrophy. The LVEF, FS, IVSFT and LVFWFT were markedly reduced and LVESV markedly increased, suggesting severe global systolic dysfunction.

### QRT-PCR

**Figure 1:**
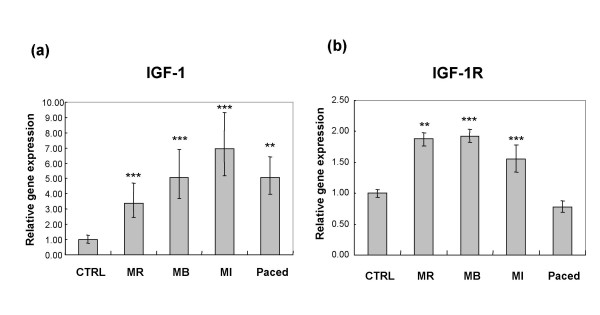
**Relative gene expression of IGF-1 (A) and IGF-1R (B) in control dogs (CTRL) vs infarcted dogs in 3 myocardial zones (MR = remote zone, MB = border zone and MI = infarct zone) and paced dogs**.

**Figure 2:**
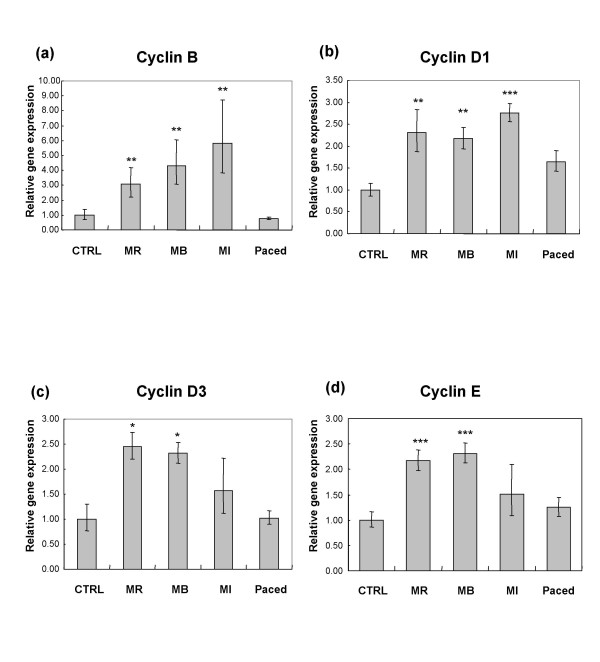
**Relative gene expression of Cyclin B (A), Cyclin D1 (B), Cyclin D3 (C) and Cyclin D3 (D) in control dogs (CTRL) vs infarcted dogs in 3 myocardial zones (MR = remote zone, MB = border zone and MI = infarct zone) and paced dogs**.

**Figure 3:**
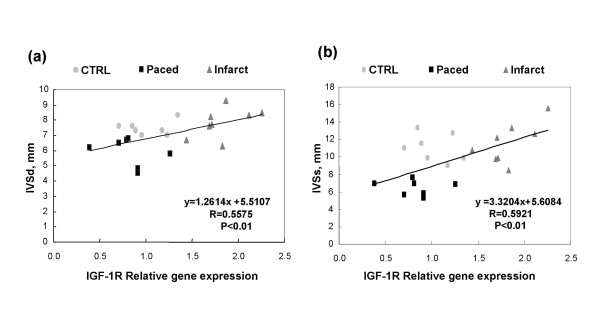
**Scatterplots of IGF-1R relative gene expression on x-axis versus IVSd (interventricular septum thickness in diastole)**. (A) and IVSs (interventricular septum thickness in systole) (B) on y-axis in control dogs (CTRL), infarcted dogs (mean expression of the 3 zones) and paced dogs.

**Figure 4:**
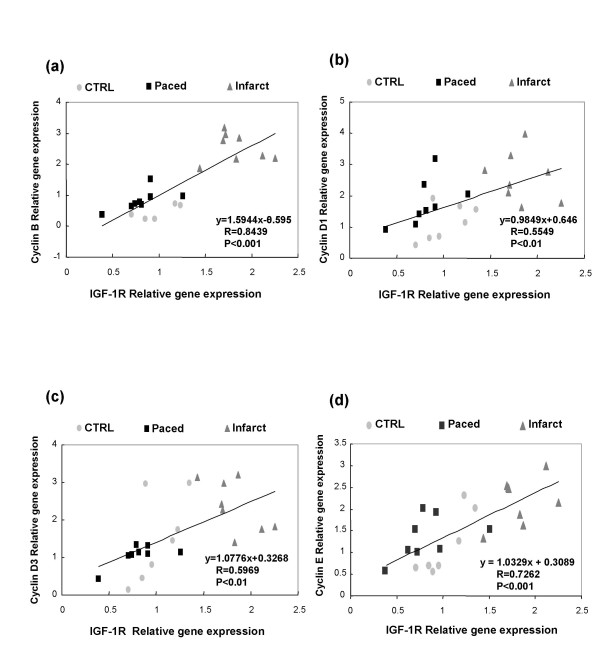
**Scatterplots of IGF-1R relative gene expression (x-axis) versus Cyclin B relative gene expression (A), Cyclin D1 relative gene expression (B), Cyclin D3 relative gene expression (C) and Cyclin E relative gene expression (D) (y-axis) in control dogs (CTRL), infarcted dogs (mean of the 3 zones) and paced dogs**.

In control dogs, the expressions of IGF-1, IGF-1R, TGFβ and cyclins A, B, D1, D2, D3 and E were not different in the left ventricular free wall from those in the interventricular septum. Therefore, in comparisons, we used the mean of the ΔCt obtained in the 2 localisations. The expression of IGF-1 was increased in the remote, border and infarction zones in dogs with ischemic cardiomyopathy, and also increased in the myocardium of dogs with tachycardiomyopathy (Figure 1a). On the other hand, the expression of IGF-1R was increased in the 3 infarction zones of the dogs with ischemic cardiomyopathy, but not in the myocardium of dogs with tachycardiomyopathy (Figure 1b). In the dogs with ischemic cardiomyopathy but not in those with tachycardiomyopathy, there were increased expressions of cyclins B, D1, D3 and E (Figure 2). The expressions of TGFβ, cyclin A and D2 remained unchanged in both models (data not shown). There were significant correlations between relative gene expression of IGF-1R and the echocardiographic variables IVSd and IVSs (Figure 3) as well as between IGF-1R and cyclin B, D1, D3 and E (Figure 4).

## Discussion

The present results show that the expression of IGF-1 is increased in 2 models of cardiomyopathy with markedly different hypertrophic responses. However, the expression of its receptor IGF-1R was increased in post myocardial infarction hypertrophic cardiomyopathy only. Furthermore, the expression of IGF-1R was related to the expression of cell cycle regulatory molecules cyclins B, D1, D3 and E, and with echocardiographic measures of myocardial hypertrophy. Therefore, these data are in keeping with the notion of a positive interaction between IGF-1 and cyclin signaling systems in the myocardial hypertrophic responses to insults.

The expression of IGF-1 has been shown to be increased in a variety of cardiac conditions [1], including acromegalic cardiomyopathy [20], chronically increased afterload [1,21] or volume overload [22] and acute myocardial infarction [4,23]. The present results add different models of chronic heart failure to this list. Thus increased gene expression of IGF-1 appears to be a universal and non-specific response of myocardial tissue to injury. The expression of IGF-1R has been shown to be increased in the left ventricle of rats with hypertrophic cardiomyopathy secondary to norepinephrine infusion [24] and also in acute myocardial infarction [4,23,25]. Furthermore, the expression of IGF-1R has been shown to decrease with ageing in the male rat heart and associated with cardiac decompensation [26]. A decreased activation of cardiomyocyte IGF-1R by IGF-1 has been reported in a model of alcoholic cardiomyopathy and this was associated with an absence of protein synthesis after IGF-1 incubation [27]. In the present study, there was an increased expression of IGF-1R in ischemic cardiomyopathy but not in tachycardiomyopathy, indirectly suggesting that parallel increased expressions of IGF-1 and its receptor are only to be observed in myocardial injury with a hypertrophic response.

Recent report of a physiological 1-0.5% turnover in adult human cardiomyocytes [9] has reactivated the question whether myocardiac hypertrophic responses might be associated with a proliferative reaction like tissue repair. We therefore wondered about possible interactions between IGF-1 signaling and the expression of cyclins, which are regulatory molecules controlling cell cycle progression [12]. Cyclins D and E regulate the synthesis of mRNA and proteins necessary for DNA synthesis and transition from the first gap G1 phase to the G1-S phase that precedes the progression to the second gap G2 phase during which additional mRNA and protein are synthesized in preparation to cell division (G2-M phase). Cyclin A is involved in the progression through the G1-S phase, and cyclin B promotes transition from the the G2 phase into mitosis [12].

In vitro, IGF-1R and its ligand regulate the re-entry of adult ventricular myocytes into the cell cycle [10]. On the other hand, cardiac myocytes proliferation slows down and ceases early during postnatal development [28] and this attenuation is paralleled by a downregulation of the myocardial IGF-1 system [29]. Fetal cardiomyocytes express high levels of cyclins both at mRNA and protein levels but shortly after birth, their expression profile becomes progressively downregulated [30]. However, the adult heart retains the ability to undergo cyclin modulation-associated DNA synthesis following hemodynamic overload or injury [11]. In a model of pressure overload-induced left ventricular hypertrophy, a transient upregulation of cyclins D2 and D3 was observed during the compensated hypertrophic stage [31]. Acute myocardial infarction is also coupled with an activation of cardiac cyclins [32]. In this study, we show that the gene expressions of cyclins B, D1, D3 and E are increased in ischemic cardiomyopathy in relation to increased IGF-1 and IGF-1R expressions and echocardiographic measures of wall thickness but are not increased in tachycardiomyopathy. It is to be noted that the expression of cyclin D2 did not vary in either model, which finding is at variance with a previous report [31]. Our data do not offer an explanation for this apparent discrepancy. We speculate that there may be differential expression of various D-type cyclins depending on the type of normal or pathologic cells [33,34], and also time-, disease- and species-related differences, not to mention D-type cyclin redundancies [35]. The observation that an infarct regression has been seen in mice overexpressing cyclin D2, but not in mice overexpressing cyclin D1 or cyclin D3 makes D-type cyclin redundancies less likely [35]. The molecular nature of the interactions between IGF-1 and cyclins and whether this implies a potential for tissue regeneration through cardiac myocyte proliferation in addition to hypertrophy remain unresolved issues.

In the present study, the gene expression of TGFβ was unchanged in the 2 types of cardiomyopathies as compared to the controls. The overexpression of TGFβ in transgenic mice results in cardiac hypertrophy which is characterized by both interstitial fibrosis and hypertrophic growth of cardiac myocytes [36]. The expression of TGFβ has been previously shown to be increased in animal models of cardiac pressure overload and infarction [37,38]. The absence of a significant increase in the expression of TGFβ in our dogs with ischemic cardiomyopathy may be related to small numbers of animals (type II error) or to different stages of the disease from those previously reported. An important limitation of the present findings is the absence of protein measurements related to mRNA content measurements. We were unable to find satisfactory antibodies enabling these measurements to be made in canine myocardial tissue. Another limitation is the absence of functional evaluations.

## Conclusion

Taken together, these results support the notion that modulation of the expression of IGF-R1 and cyclins B, D1, D3 and E genes are involved in the various hypertrophic response observed in cardiomyopathies.

## Competing interests

The authors declare that they have no competing interests.

## Authors' contributions

MMah carried out the molecular study. MMat, KT, AM and KM participated in the intact animal experiments. IH helped in the molecular study. KM performed echocardiographies. MMah, MMathieu, KT, KT, IH, AM, RN and KM participated in the discussions of the experimental design, the interpretation of the results and the successive drafts of the present report. All authors read and approved the final manuscript.

## Pre-publication history

The pre-publication history for this paper can be accessed here:


